# Supplementary material for: Laminarin Attenuates ROS-Mediated Cell Migration and Invasiveness through Mitochondrial Dysfunction in Pancreatic Cancer Cells
Source: Antioxidants (Basel). 2022 Aug 30;11(9):1714. doi: 10.3390/antiox11091714 (PMC9495390; doi:10.3390/antiox11091714)
Supplement: Supplementary file 1 [file antioxidants-11-01714-s001.zip › antioxidants-1883996-supplementary.pdf]

Table S1. The information about antibodies we used.

Antibody	Catalog Number	Company	Dilution
p-AKT (Ser473)	4060	Cell Signaling Technology	1:1000
AKT	9272	Cell Signaling Technology	1:1000
p-ERK1/2 (Thr202/Tyr204)	9101	Cell Signaling Technology	1:1000
ERK1/2	4695	Cell Signaling Technology	1:1000
p-JNK (Thr183/Tyr185)	4668	Cell Signaling Technology	1:1000
JNK	9252	Cell Signaling Technology	1:1000
p-P38 (Thr180/Tyr182)	4511	Cell Signaling Technology	1:1000
P38	9212	Cell Signaling Technology	1:1000
KRAS	91054	Cell Signaling Technology	1:1000
β-actin	sc-47778	Santa Cruz Biotechnology	1:1000

Table S2. The primers we used in quantitative real-time PCR

Gene	Size (bp)	GenBank accession No.	Primer Sequence (5'→3')
forkhead box protein M1 (FOXM1)	104	NM_001243088.2	Forward : AGTCACACCCTAGCCACTGC<br>Reverse : ACCATTGCCTTTGTTGTTCC
vascular endothelial growth factor A (VEGFA)	109	NM_001025366.3	Forward : CTGCTCTACCTCCACCATGC<br>Reverse : AGCTGCGCTGATAGACATCC
C-X-C motif chemokine ligand cadherin-1 (CDH1)	120	NM_001317184.2	Forward : GAACACATTTGCCCAATTCC<br>Reverse : CCCCTACCCCTCAACTAACC
tissue inhibitors of metallopeptidase1 (TIMP1)	109	NM_003254.3	Forward : CCTCCAAGGCTCTGAAAAGG<br>Reverse : CAGGATTCAGGCTATCTGGG
glyceraldehyde-3-phosphate dehydrogenase (GAPDH)	149	NM_001256799.3	Forward : GGCTCTCCAGAACATCATCC<br>Reverse : TTTCTAGACGGCAGGTCAGG
